# Correction: Probing intrinsic dynamics and conformational transition of HIV gp120 by molecular dynamics simulation

**DOI:** 10.1039/d0ra90094j

**Published:** 2020-10-07

**Authors:** Yi Li, Xiao-Ling Zhang, Xue Yuan, Jiang-Chun Hou, Peng Sang, Li-Quan Yang

**Affiliations:** College of Mathematics and Computer Science, Dali University Dali China; Science and Education Department, Second People’s Hospital of Yunnan Province Kunming China; College of Agriculture and Biological Science, Dali University Dali China ylqbioinfo@gmail.com

## Abstract

Correction for ‘Probing intrinsic dynamics and conformational transition of HIV gp120 by molecular dynamics simulation’ by Yi Li *et al.*, *RSC Adv.*, 2020, **10**, 30499–30507, DOI: 10.1039/d0ra06416e.

The authors regret that they incorrectly quoted the structure template reference as 5FUU. This should have been 3J70.

Therefore, the authors would like to make the following corrections:

The first sentence of Section 2.1 currently contains the text “Protein Data Bank (PDB, http://www.rcsb.org) with accession IDs are 5FYK^12^ and 5FUU^13^ at 3.11 Å and 4.19 Å resolution, respectively.” This should read “Protein Data Bank (PDB, http://www.rcsb.org) with accession IDs 5FYK^12^ and 3J70 at 3.11 Å and 20 Å resolution, respectively.”

The sentence in section 2.1 that reads “The full-length amino acid sequence of these two gp120 structures is the HIV JR-FL strain 14 which was also used in the smFRET experiment.” should read “The full-length amino acid sequence of closed gp120 structures is the HIV JR-FL strain 14 which was also used in the smFRET experiment.

The caption for Fig. 1 currently contains the text “The structural models of gp120. (A and B) Ribbon representation of the closed and open gp120 with PDB IDs of 5FYK and 5FUU, respectively.” This should read “The structural models of gp120. (A and B) Ribbon representation of the closed and open gp120 with PDB IDs of 5FYK and 3J70, respectively.”

Ref. 13 should not have been included in the reference list as it corresponds to the incorrect structure template reference.

The Royal Society of Chemistry apologises for these errors and any consequent inconvenience to authors and readers.

## Supplementary Material

